# Effect of long-term treatment with classical neuroleptics on NPQ/spexin, kisspeptin and POMC mRNA expression in the male rat amygdala

**DOI:** 10.1007/s00702-018-1868-2

**Published:** 2018-02-27

**Authors:** Artur Pałasz, Marcelina Pałka, Łukasz Filipczyk, Itiana Castro Menezes, Ewa Rojczyk, John J. Worthington, Aneta Piwowarczyk-Nowak, Marek Krzystanek, Ryszard Wiaderkiewicz

**Affiliations:** 1 Department of Histology, School of Medicine in Katowice, Medical University of Silesia, ul. Medyków 18, 40-752 Katowice, Poland; 2 Department of Neurosciences and Behaviour, Faculty of Medicine, University of São Paulo, Av. Bandeirantes 3900, Ribeirão Preto, São Paulo 14049-900 Brazil; 3 Department of Descriptive and Topographic Anatomy, School of Medicine with Division of Dentistry in Zabrze, Medical University of Silesia, ul. Jordana 19, 41-808 Zabrze, Poland; 4 Division of Biomedical and Life Sciences, Faculty of Health and Medicine, Lancaster University, Lancaster, LA1 4YQ UK; 5 Department of Anatomy, School of Medicine in Katowice, Medical University of Silesia, ul. Medyków 18, 40-752 Katowice, Poland; 6 Department and Clinic of Psychiatric Rehabilitation, School of Medicine in Katowice, Medical University of Silesia, ul. Ziolowa 45/47, 40-635 Katowice, Poland

**Keywords:** Amygdala, Spexin, Kisspeptin, POMC, Chlorpromazine, Haloperidol

## Abstract

Neuroleptics modulate the expression level of some regulatory neuropeptides in the brain. However, if these therapeutics influence the peptidergic circuits in the amygdala remains unclear. This study specifies the impact profile of the classical antipsychotic drugs on mRNA expression of the spexin/NPQ, kisspeptin-1 and POMC in the rat amygdala. Animals were treated with haloperidol and chlorpromazine for 28 days prior to transcript quantification via qPCR. Haloperidol and chlorpromazine induced a change in the expression of all neuropeptides analyzed. Both drugs led to the decrease of Kiss-1 expression, whereas in POMC and spexin/NPQ their up-regulation in the amygdala was detected. These modulating effects on may represent alternative, so far unknown mechanisms, of classical antipsychotic drugs triggering pharmacological responses.

## Introduction

The pharmacology of antipsychotic drugs belongs to the important fields of contemporary neurophysiology and applied psychiatry. Accumulating, but still very limited amount of data suggests, that neuroleptics may directly and/or indirectly affect the peptidergic signaling circuits within various neuronal assemblies. Chronic haloperidol administration causes a decrease in NPY mRNA expression in the rat amygdala and hippocampus, while clozapine and olanzapine exert the same effects in the striatum, nucleus accumbens and anterior cingulated cortex (Huang et al. 2006). Both chlorpromazine and clozapine modulate CRH expression, probably via activation of the PI3K/Akt signaling cascade, although the involvement of the protein kinase C (PKC) pathway is also suggested (Basta-Kaim et al. 2006). Antipsychotics also differentially modulate signaling pathways, with haloperidol elevating CRH and GnRH in the rat hypothalamus (Park et al. 2011), while quetiapine and olanzapine inhibit CRH release in organotypic slices of rat hypothalamus and hippocampus (Tringali et al. 2009). Furthermore, haloperidol increases, but risperidone decreases neurotensin levels in the rat striatum, hippocampus and frontal cortex (Gruber et al. 2002). Decreased NUCB2/nesfatin-1 expression is reported in the rat hypothalamus after long-term haloperidol administration (Rojczyk et al. 2015). It has also been reported that olanzapine increases calcitonin gene-related peptide (CGRP) in the rat brain (Angelucci et al. 2008).

The amygdaloid complex is formed by nuclei with heterogeneous anatomical and functional characteristics (de Olmos et al. 2004; Rasia-Filho et al. 2000), integrating endocrine, sympathetic/parasympathetic, behavioral activities, emotion processing, facial expression recognition and feeding behavior (Janak and Tye 2015; Quagliotto et al. 2015). These combined functions, as well as amygdala plasticity (Becker et al. 2013), may be altered by the action of neuropeptides (Chung and Moore 2007, 2009; Debiec 2005; Knobloch et al. 2012; Kritman et al. 2017; Quagliotto et al. 2015).

The administration of neuroleptics as the dopamine receptor antagonists leads to modulation of diverse neuronal populations in the amygdala implicating several shared effects of these drugs. A recent report suggests that stabilized dopamine release in the amygdala is an important mechanism of haloperidol and clozapine actions during emotional processing (Kawano et al. 2016). The D_1_ family receptors (D_1_R) seems to play a crucial role the functioning of the amygdala and their activation in the basolateral nucleus (BLA) is necessary for the fear related responses (Li and Rainnie 2014; Macedo et al. 2007) e.g. after direct injections of D_1_R antagonists into this structure the anxiolytic effects occured (de la Mora et al. 2005). The involvement of peptidergic circuits underlying the pharmacological activity of antipsychotic drugs remains unclear. Numerous neuropeptide releasing amygdalar neurons have a distinct expression of D_1_ and D_5_ dopamine receptors (Muly et al. 2009). It does suggest that antipsychotic drugs may control peptidergic signaling via blockage of dopamine transmission. Both haloperidol and clozapine activate dynorphinergic (DYN) but not enkephalinergic (ENK) GABA neurons in central amygdaloid nucleus (Ma et al. 2003). The expression of NPY mRNA in the rat amygdala decreased after short term treatment with haloperidol but the NPY-peptide level remained unaffected. Conversely, an extended, 14-day haloperidol administration induced an elevation in NPY mRNA expression (Śmiałowska et al. 2001). Short term treatment with haloperidol decreased the number of neurotensin-expressing neurons in the rat medial amygdala (Eggerman and Zahm 1988). Noteworthy, haloperidol administration may also modulate the functional integration in the neural network nodes linked to the amygdala (Haaker et al. 2016).

The peptide derived from the NPQ/spexin (SPX) precursor contributes to CNS-mediated control of arterial blood pressure, salt and water balance and modulates nociceptive responses. As well, SPX seems to modulate food-intake and reproduction, acting as a potent anorexigenic factor. Numerous SPX immunopositive neurons have been described in the animal brain, with the highest reaction detected in the hypothalamus. Moderate SPX immunoreactivity has been found in the hippocampus, amygdala, cerebellum and brainstem (Porzionato et al. 2010). A recent study suggests that SPX is an alternative endogenous ligand for the galanin receptors GALR2/3, fear response mediators (Lu et al. 2008), and exhibit even higher affinity toward GALR3 than galanin (Kim et al. 2014). SPX mRNA expression was downregulated in the amygdala and hypothalamus and upregulated in hippocampus, striatum and cerebellum after chronic administration of escitalopram in adult male rats, suggesting that SPX may influence food-intake control, anxiety responses and the HPG axis (Palasz et al. 2016b). Despite these preliminary studies, currently there is no literature regarding SPX action in the amygdaloid complex.

Another regulatory peptide kisspeptin plays an important role in reproduction (Stephens and Kauffman 2017) and high concentrations are required to modulate neuronal activity (Liu and Herbison 2016). Kisspeptin signaling regulates the ovarian cycle through the control of GnRH synthesis in the hypothalamus. At present, little is known about the potential role of kisspeptin outside of the hypothalamus, although Kiss-1 mRNA expression has been found in the hippocampus and kisspeptin neurons have been also detected in the medial amygdala and the bed nucleus of stria terminalis (Gottsch et al. 2004; Xu et al. 2012). The expression of Kiss-1 is predominantly in the medial nucleus of the amygdala (MeA), a sexual dimorphic area (Newman 1999) known to be related to social, emotional and sexual behaviors (Kim et al. 2011; Rasia-Filho et al. 2000; Stephens and Kauffman 2017). The presence of sex steroids may raise the expression of Kiss-1 in the MeA of male and female rats, with Kiss-1 expression dependent on the estrous cycle, occurring through estrogen receptors, in females (Kim et al. 2011). Corroborating the data that Kiss-1 expression is important for reproduction, Kiss-1 expression was absent in postnatal amygdala, suggesting that it may be expressed only in age near puberty (Cao and Patisaul 2013). Furthermore, Comninos et al. (2016) found that kisspeptin signaling within MeA contributes to the modulation of gonadotropin release and pulsatility. Pineda et al. (2017) assessed the localization of kisspeptin neurons in the amygdala and their innervation by the dopamine and vasopressin systems.

POMC neurons play a central role in regulating body weight and stress related responses (Palasz et al. 2016a). POMC is a polyprotein which expression is present mainly in the hypothalamus and pituitary, where it is processed into the neuropeptides—corticotropin, lipotropins, melanotropins, and endorphins (Chretien and Mbikay 2016). Although expression of the POMC gene and protein seems to be restricted to hypothalamic nuclei, intriguingly lower POMC mRNA levels were also detected in the limbic structures including the amygdala and hippocampus (Bai et al. 2005; Civelli et al. 1982; Niikura et al. 2013) that suggest an uninvestigated role in stress and affective responses. The hypothalamic POMC neurons may mediate reward and analgesia sending their long afferents to the periaqueductal gray, ventral tegmental area (VTA), amygdala and dorsal vagal complex (King and Hentges 2011).The MC4R melanocortin receptor was distributed through all nuclei of the amydaloid complex of adult rat brain and is congruent to central melanocortin systems regarding autonomic regulation and feeding control (Kishi et al. 2003). There are direct inputs from MeA and amygdaloid and hippocampal areas to POMC neurons in the arcuate nucleus and inputs from the medial part of central amygdaloid nucleus to POMC neurons in the nucleus tractus solitarius (Wang et al. 2015).

At present, there is still insufficient information dealing with the influence of neuroleptics on peptidergic signaling in various brain regions. Particularly, a large knowledge gap exists concerning newly discovered neuropeptides such as SPX and kisspeptin. Thus, the main purpose of the presented study was to estimate how classical neuroleptics chlorpromazine and haloperidol affect the NPQ/spexin, kisspeptin-1 and POMC mRNA level in the rat amygdala.

## Materials and methods

Studies were carried out on adult (5 months old) male Sprague–Dawley rats. All experimental procedures were approved by the Local Bioethical Committee (agreement no. 36/2012) and were conducted in a manner consistent with NIH Guidelines for Care and Use of Laboratory Animals. Three groups of animals received chlorpromazine (5 mg/kg/day), haloperidol (2 mg/kg/day) or control saline vehicle by intraperitoneal injection for 4 weeks. 24 h after the last drug administration, rats were quickly anaesthetized with isoflurane and sacrificed. Total mRNA was extracted from microsurgically excised whole amygdalae via the phenol–chloroform method using Trizol™. Collected mRNA samples were transcribed into cDNA during incubation in buffered solution of reverse transcriptase MMLV-RT with RNAsin, oligo-dT and mix of nucleotides at 42 °C for 60 min using DNA Thermal Cycler 480 (Perkin Elmer Inc., Waltham, MA). After that, Quantitative Real-Time PCR reaction (qPCR) was performed by FastStart SYBR Green Master (Roche) in a Light Cycler 1 96 (Roche) thermal cycler 32 rounds; 1 min at 94 °C 1 min at 65 °C (for Kiss-1) or 55.8 °C (for POMC) and finally 90 s at 72 °C. Beta-2-microglobulin (B2m) was chosen as a standard internal reference gene. Primer sequences; B2m: forward: 5′-CGAGACCGATGTATATGCTTGC-3′, reverse: 5′-GTCCAGATGATTCAGAGCTCCA-3′, POMC: forward: 5′-CCAAGCGCTCCA CGAGACTT, reverse: 5′-TTGGGAGCAGGTACCCTC; Kiss-1: forward 5′-TGGCACCTGTGGTGAACCCTGAAC-3′, reverse: GCCACCTGCCTCCTGCCGT AGCGC For NPQ/spexin analysis, cDNA was amplified using the TaqMan Gene Expression Assay Spexin (Rn01749065_m1, Applied Biosystems) and TaqMan Gene Expression Master mix (4369016, Applied Biosystems). Optimal hybridization temperature was established according to a gradient PCR and was 50 and 59 °C. Statistical analyses were performed using Statistica (Systat Software). Data are presented as mean ± SEM. Mean differences between experimental groups were analyzed using one-way ANOVA followed by Tukey’s post hoc test. Differences were considered statistically significant at *p* ≤ 0.01.

## Results and discussion

**Fig. 1:**
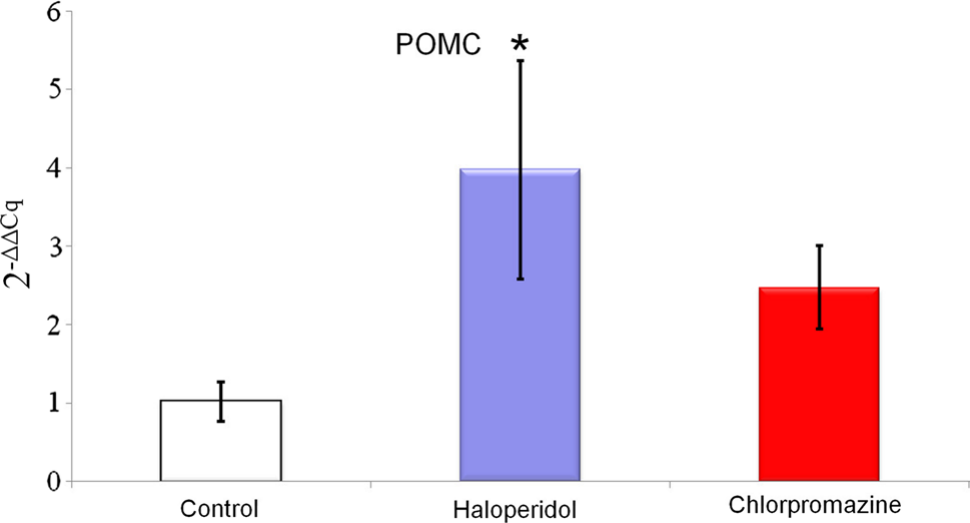
Relative mRNA expression of NPQ/spexin in the rat amygdala after chronic (every day for 4 weeks) chlorpromazine and haloperidol administration. Beta-2-microglobulin (B2m) was used as a reference gene. Values are expressed as mean ± SEM. One-way ANOVA followed by Tukey’s HSD post hoc test was used for statistical analysis (experimental group vs control). *p* ≤ 0.01 is considered as statistically significant (asterisk)

**Fig. 2:**
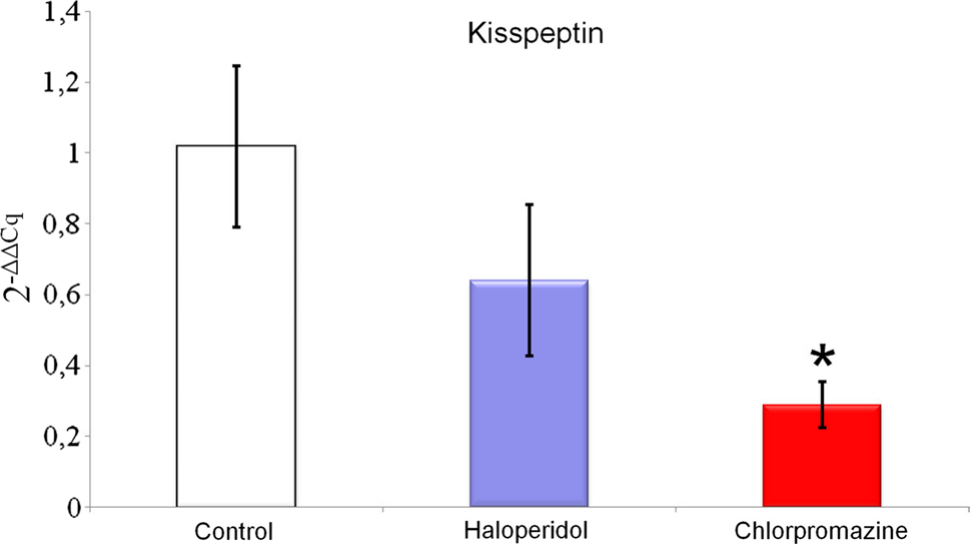
Relative mRNA expression of proopiomelanocortin (POMC) in the rat amygdala after chronic (every day for 4 weeks) olanzapine administration. Beta-2-microglobulin (B2m) was used as a reference gene. Values are expressed as mean ± SEM. One-way ANOVA followed by Tukey’s HSD post hoc test was used for statistical analysis (experimental group vs control). *p* ≤ 0.01 is considered as statistically significant (asterisk)

**Fig. 3:**
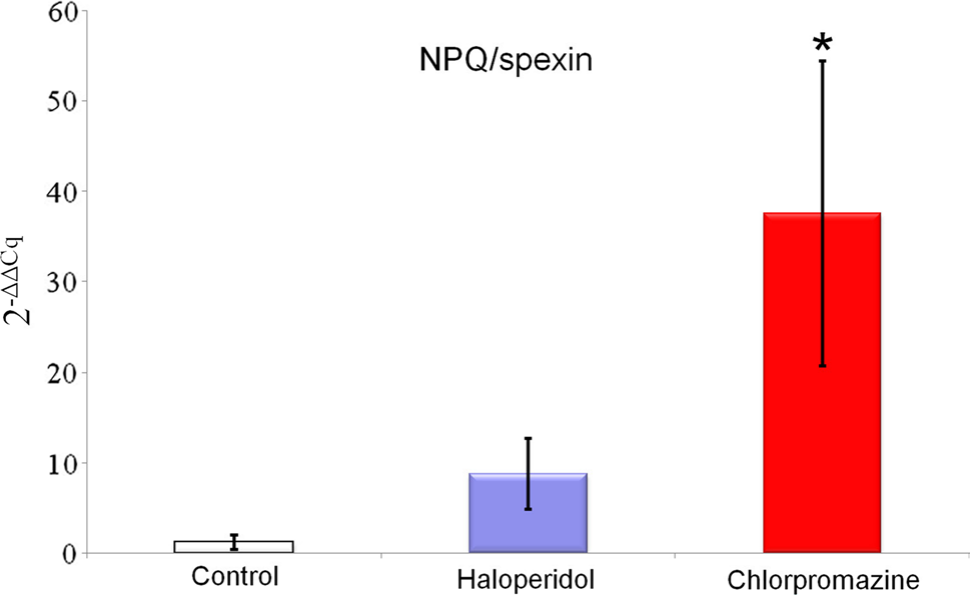
Relative mRNA expression of kisspeptin in the rat amygdala after chronic (every day for 4 weeks) olanzapine administration. Beta-2-microglobulin (B2m) was used as a reference gene. Values are expressed as mean ± SEM. One-way ANOVA followed by Tukey’s HSD post hoc test was used for statistical analysis (experimental group vs control). *p* ≤ 0.01 is considered as statistically significant (asterisk)

Chronic treatment with haloperidol and chlorpromazine increased NPQ/spexin mRNA expression in the rat amygdala (haloperidol 8.760 ± 3.937; chlorpromazine 37.540 ± 16.814 vs control 1.211 ± 0.807; Fig. 1), but this effect was statistically significant only for chlorpromazine. A similar effect was observed for POMC mRNA expression in the rat amygdala—it was increased after long-term administration of both drugs (haloperidol 3.983 ± 1.391; chlorpromazine 2.478 ± 0.528 vs control 1.021 ± 0.256; Fig. 2), However, this time, statistical significance was obtained exclusively for haloperidol. Rats treated chronically with haloperidol and chlorpromazine manifested decreased kisspeptin mRNA expression in amygdala (haloperidol 0.640 ± 0.212; chlorpromazine 0.289 ± 0.065 vs control 1.017 ± 0.228; Fig. 3), which was statistically significant only for chlorpromazine. The influence of long-term treatment with two typical neuroleptics on NPQ/SPX, Kiss-1 and POMC transcript expression in the rat amygdala were assessed. Although the clinical applications of chlorpromazine and haloperidol are currently limited, they are still a relevant model in basic pharmacological studies. In the present study we found, that both the antipsychotics affected them RNA expression of the analyzed neuropeptides, where chlorpromazine was characterized by the generally strongest modulatory effect. After chronic treatment with this neuroleptic a significant increase of SPX mRNA level was noted. Furthermore, haloperidol caused a similar elevation of SPX mRNA expression; however its effect was certainly lower than chlorpromazine treatment. Due to a lack of previous pharmacological studies on SPX and existence of substantial structural differences between SPX and other regulatory peptides, the results obtained are currently difficult to interpret. In fact, just one article dealing with SPX expression after the administration of neuropsychiatric drugs is available in the literature (Palasz et al. 2016b). Our previous finding shows that extended treatment with escitalopram increases both SPX mRNA and protein expression in the rat hippocampus and striatum, but decreases expression in the hypothalamus. The differences observed in studied drugs are most likely a consequence of different modes of their action at the molecular level Hypothetically, an increase of SPX expression after neuroleptic treatment may be associated with the decreased level of anxiety in rats, but this possibility has to be examined in further behavioural studies. Remarkably, the high increase of SPX mRNA level after chronic chlorpromazine administration seems to be analogical to previous data showing that expression of neuropeptide S (NPS) mRNA in the hypothalamus of rats treated with olanzapine was also elevated (Palasz and Rojczyk 2015). Chlorpromazine as well as haloperidol gives rise to increased POMC expression, with seven-times higher modulation by haloperidol than chlorpromazine. The similar trend was reported in other experiments, where the effects of long-term treatment (28 days) with both first and second-generation neuroleptics on POMC gene expression were evaluated (Rojczyk et al. 2015). Despite a relatively limited increase of atypical olanzapine expression, significant statistical changes were observed as well as typical haloperidol expression compared to control. After chronic treatment with chlorpromazine, POMC and Y_1_ receptor mRNA expression increased, corresponding with the submitted results. Another experiment with long-term treatment with olanzapine shows increased POMC mRNA levels in the male rat amygdala (Palasz et al. 2016a).Our data are approximate to this study for kisspeptin-1 after chronically administered haloperidol, but the difference was noted with olanzapine increasing POMC expression, on the other hand haloperidol caused decrease Kiss-1 expression. Our results may also correlate with experiments performed by Kursungoz et al. (2015), where extended (4 weeks) treatment with risperidone decreased the POMC, NPY/AgRP mRNA levels in the rat hypothalamus, while increasing CART mRNA expression. Interestingly, our study revealed an inhibitory effect of both haloperidol and chlorpromazine on Kiss-1 expression in the amygdala. This may cautiously suggest an existence of alternative pharmacological model of neuroleptics’ action in the limbic system with unknown neurophysiological consequences. A significant decrease of the Kiss-1 mRNA level in the amygdala corresponds with the previous finding showing the same effect in the rat hypothalamus and after long-term treatment with haloperidol, chlorpromazine and olanzapine (Palasz et al. 2017). Our results are also in line with other reports, that haloperidol decreased NPY mRNA expression in the rat amygdala and hippocampus, while olanzapine produces the same effect in the nucleus accumbens, striatum and cingulate cortex (Huang et al. 2006). Interestingly, both NPY and nesfatin-1 mRNA expression in the rat hypothalamus were also decreased after chronic exposure to neuroleptics (Rojczyk et al. 2015). On the other hand our results contradict findings showing that haloperidol supports the NPY mRNA expression in the rat amygdala (Śmiałowska et al. 2001) and increase hypothalamic GnRH and CRF mRNA levels (Park et al. 2011; Umathe et al. 2009).The changes in Kiss-1 mRNA expression seem to be especially interesting in the context of recent evidence proving an important role of kisspeptin signaling in the physiology of medial amygdala (Di Giorgio et al. 2014; Xu et al. 2012; Yeo et al. 2016). Taking into account that Kiss-1 expression in the amygdala is controlled by circulating sex hormones (Cao and Patisaul 2013; Kim et al. 2011), and kisspeptin neurons in MeA are sensitive to the pheromone molecules (Pineda et al. 2017), the analogous comparative study on females has to be definitely proven. A previously mentioned study shows that long-term treatment with olanzapine, haloperidol and chlorpromazine modulated Kiss-1 mRNA expression in the rat hypothalamus (Palasz et al. 2017). A different pattern of alterations in the kisspeptin expression between amygdala and hypothalamus may be explained by their diverse cellular composition and receptor profile. It should not be excluded that a decrease in kisspeptin signaling after neuroleptic administration may evoke some subtle anxiety-related behavioural changes in animals. This may suggest an alternative outcome of central neuroleptics’ action. Taking together, both haloperidol and chlorpromazine affect kisspeptin-1 mRNA expression in the rat amygdala, suggesting Kiss-1 participation in the pharmacological effects of this drug at the level of limbic structures. Long-term chlorpromazine administration increases the level of spexin/NPQ mRNA expression in the amygdala, throwing a new light on the physiology of this previously poorly studied, but promising regulatory neuropeptide. Haloperidol modulates POMC expression in the amygdala, which may be one of the unknown, alternative mechanisms of its action in the CNS. Modulatory impact on POMC, spexin/NPQ and kisspeptin-1 expression may be one of the potential, so far unknown mechanisms of pharmacological effects triggered by classical antipsychotic drugs in animal models. Although both chlorpromazine and haloperidol are antipsychotic drugs with non-specific affinity to dopaminergic receptors they affected the neuropeptide mRNA expression in a different manner. The reasons for this discrepancy are unclear, however, we can suggest two explanations why these changes were perceived. It should pointed out firstly that these typical neuroleptics have a different molecular structure, haloperidol belongs to the butyrophenone family but chlorpromazine is a phenothiazine derivative. More importantly haloperidol, a potent D_2_ inverse agonist may have a different antagonistic affinity to amygdalar D_1_ and D_5_ receptors than chlorpromazine.

Patients with schizophrenia may have impaired activity of the amygdala as implicated by decreased emotion perception but augmented arousal associated with psychotic symptoms (Rasetti et al. 2009; Aleman and Kahn 2005). A study by Blasi et al. (2009) shows that long-term treatment with olanzapine may be associated with specific changes in activity of the amygdala and prefrontal cortex during emotional processing in schizophrenia. The potential role of amygdalar neuropeptides in the pathophysiology of mental disorders is almost unknown. An interesting report suggests that social cognitive deficits in schizophrenia may be related to the abnormalities in the oxytocinergic signaling in the amygdala (Rosenfeld et al. 2011). The recent study seems to supports this hypothesis showing that in patients with schizophrenia spectrum disorders (SCZ) the oxytocin receptor gene polymorphisms were associated with low amygdala activation (Haram et al. 2016). Furthermore, the number of somatostatin-expressing neurons was decreased in the amygdala of patients with schizophrenia (Pantazopoulos et al. 2017). On the other hand, an interesting study by Majercikova and Kiss (2015) shed surprising light on the matter of antipsychotic action at the level of peptidergic pathways in the rat amygdala. An effect of atypical neuroleptic asenapine on FosB/ΔFosB expression and activity of CRH-expressing neurons in the central amygdalar nucleus (CeA) under the condition of chronic unpredictable variable mild stress (CMS) were examined. Unexpectedly, no statistically significant changes were observed, which suggests neither CRH-related nor Fos signaling pathways are involved in asenapine pharmacological effects in the amygdala.

Neuropeptide signaling contribution in amygdala function is a refined issue in existing references, especially in terms of the pharmacological aspect. This study focuses on analysis of well-known and significant neuropeptide gene expression (kisspeptin and POMC) as well as the newly identified (SPX), and provides original data and hypothesis concerning activity of alternative mechanisms, using antipsychotic drugs in rat amygdala. It should not be therefore excluded that the putative pharmacomodulation of the neuropeptide signaling in the amygdala may be potentially helpful in the future therapy of certain neuropsychiatric disorders including schizophrenia. We have to point out that there are some limitations of our study, for instance the protein levels were not measured, the receptor–ligand interactions were not determined and the precise analysis of particular amygdaloid nuclei was not provided. It has to be definitely an aim of the further investigations. Undoubtedly this finding represents merely an initial introduction to forthcoming experimental works in the field of novel regulatory neuropeptides. Although any possible applications of the novel peptidergic modulators still remain in the area of speculation intensive searching for the selective agonists/antagonists of their known receptors may contribute to opening of a new chapter in the meaning and treatment of mental disorders.
